# A comparative study on indicators of vitamin A status and risk factors for sensitivity and specificity of the methods to detect vitamin A deficiency

**DOI:** 10.1186/s12986-023-00768-7

**Published:** 2023-11-16

**Authors:** Olivier O. Sombié, Augustin N. Zeba, Jérome W. Somé, Adama Kazienga, Michael Grahn, Sherry A. Tanumihardjo, Stefaan De Henauw, Souheila Abbeddou

**Affiliations:** 1 Unité Nutrition et Maladies Métaboliques, Institut de Recherche en Sciences de la Santé/Direction Régionale de l’Ouest, Bobo-Dioulasso, Burkina Faso; 2 Department of Public Health and Primary Care, Faculty of Medicine and Health Sciences, Ghent University, Ghent, Belgium; 3 Unité Nutrition et Maladies Métaboliques, Institut de Recherche en Sciences de la Santé/Direction Générale, Ouagadougou, Burkina Faso; 4 Department of Translational Physiology, Infectiology and Public Health, Faculty of Veterinary Medicine, Ghent University, Ghent, Belgium; 5 Department of Nutritional Sciences, University of Wisconsin-Madison, Madison, USA

**Keywords:** Sensitivity, Specificity, Total liver reserve, Serum retinol, Retinol-binding protein, Vitamin A deficiency, Children, Burkina Faso

## Abstract

**Background:**

Serum retinol (SR) and retinol-binding protein (RBP) are commonly used indicators, but they are affected by infections and inflammation. This study aimed to assess the sensitivity and specificity of VA indicators to detect vitamin A deficiency (VAD) in 36–59-month-old children living in a rural area in Burkina Faso.

**Methods:**

In a community-based study, two cross-sectional surveys were carried out from November 2016 to September 2017 in the health district of Dandé in Burkina Faso. The surveys included 115 children 36–59 months old. Indicators of VA and inflammation assessed in all children included SR, RBP and total liver VA reserves (TLR) estimated by retinol isotope dilution, and inflammation markers (C-reactive protein (CRP) and alpha 1-acid glycoprotein (AGP)). We calculated the sensitivity, specificity, positive and negative predictive values. In addition, the effects of inflammation, helminth infection, and season on sensitivity and specificity were assessed.

**Results:**

The prevalence of VAD assessed by SR (< 0.7 µmol/L), RBP (< 0.7 µmol/L), and TLR (< 0.1 µmol/g liver) were, respectively, 30.9%, 33.3%, and 0%. Compared to TLR, the specificity, positive predictive value, and negative predictive value of SR were 71.1%, 0%, and 100%, and of RBP, were 68.9%, 0%, and 100%, respectively. The sensitivity was indeterminable for SR and RBP. The specificity of SR and RBP was lower during the dry season. Elevated CRP (> 5.0 mg/L) and AGP (> 1.0 g/L) were detected in 1.9% and 28.6% of children, respectively. The adjustment of VA indicators for inflammation improved SR’s specificity to 75.9% and decreased RBP’s specificity to 67.8%.

**Conclusion:**

No cases of VAD were identified by TLR. However, (inflammation-adjusted) SR and RBP had varying accuracy in the estimation of VAD.

**Trial registration:**

The study was registered, retrospectively, on 22 March 2018 as a clinical trial with the Pan African Clinical Trials Registry under the number Cochrane South Africa; PACTR201803002999356.

## Introduction

Vitamin A deficiency (VAD) remains a public health concern that affects disproportionally vulnerable populations in low- and middle-income countries (LMICs) [1]. Several interventions, including vitamin A (VA) supplementation, food fortification and biofortification, and promotion of dietary diversity were upscaled to tackle VAD [2, 3].

Methods used to assess VA status include dietary intake assessment, medical examination of xerophthalmia (the ocular manifestations of VAD), and biochemical analyses. Biochemical methods of VA status assessment are based on organic solvent extraction of serum (retinol) or tissue samples (liver biopsy), followed by measurement using high-performance liquid chromatography equipped with UV detector at single or multiple wavelengths. Most modern methods use forms labeled with deuterium or carbon-13, commonly named retinol isotope dilution test (RID) [4]. Proteins involved in retinoid transport such as serum retinol-binding protein (RBP) and function are analyzed using spectrophotometric, immunologic, or molecular biologic methods [5]. Biomarkers of VA vary in their accuracy depending of VA status [6]. Based on total liver VA reserve (TLR) concentration, the optimum liver concentrations range between 0.1 and 1.0 µmol/g of liver (adequate), and > 1.0 µmol/g of liver reflects hypervitaminosis A for both RID and liver samples [7]. The direct measurement of liver VA stores by biopsy is the most accurate test, but this is rarely an option for obvious reasons; the test is not justified ethically and not feasible in population-based studies. RID is expensive to be a community tool to assess VA status. The relative dose response (RDR) test requires two blood samples taken 5 h apart and is usually considered impractical in large community surveys [8]. The modified RDR test does not require the baseline blood sample, but there is still a 4 to 6 h wait period. Methods that are commonly used, especially in community surveys are serum retinol and RBP concentrations. Community-based surveys assessing VA have been hampered by the complexities of assessing VA status during episodes of infection [5]. During infections, the hepatic acute phase response involves increasing or decreasing protein synthesis. Acute-phase response to infection includes an increase in C-reactive protein (CRP) and α1-acid glycoprotein (AGP) and a decrease in serum concentrations of retinol and RBP [9]. Circulating retinol concentration is additionally affected by energy intake and plasma protein concentration [4].

The World Health Organization (WHO) defines the cut off ≥ 20% of VAD in children with serum retinol concentration < 0.7 µmol/L as a severe public health problem [10]. Serum retinol is homeostatically controlled, therefore changes in its concentration are not linearly correlated with changes in total liver VA reserves, except in extremely low or high hepatic VA stores. Thus, in infection-free individuals, low serum retinol concentrations indicate very low to depleted hepatic VA stores ( < ~ 0.07 µmol total retinol/g liver). At higher hepatic VA stores, corresponding to values above 3 µmol total retinol/g liver and indicating a state of hypervitaminosis A, elevated serum retinyl ester concentrations are measurable [11]. Contrarily to serum retinol, RBP concentrations remain normal in cases of extremely increased hepatic VA stores [11]. During infections, both serum retinol and RBP concentrations decrease, independent of liver VA stores [8, 10, 12–14]. Methods estimating TLR remain the most accurate in assessing VA status in a range of VA concentrations and in case of infections and inflammations [8, 15]. The aforesaid indicates clearly that serum retinol and RBP are poor indicators of VA status, and their values become questionable during infections [16]. Thurnham and colleagues developed a method to adjust, in surveys, the values of serum retinol and RBP concentrations, considering the indicators of inflammation CRP and AGP [13]. Wessells and colleagues found that malaria infection affects retinol concentrations even after adjusting for inflammation indicators [17]. In this context, the purpose of this study was to assess the sensitivity and the specificity of serum retinol and RBP concentrations in detecting VAD against TLR using retinol isotope dilution technique (RID), with and without adjustment for inflammation.

## Materials and methods

### Study area and subjects

The present analysis was carried out in the context of a study entitled “Vitamin A status in children of 36–59 months of age in a malaria-endemic rural area in Burkina Faso”, which aimed to assess and monitor VA status of preschool children susceptible to infections. This study was conducted in two villages, Sourkoudougou and Banakeledaga, located at the health district of Dandé, in South West Burkina Faso. Dandé district is at a distance of 30 Km from Bobo Dioulasso, the second largest city in the country. The region is known for its agricultural activity that benefits from the Soudanian climate zone. During the rainy season, that lasts between May and September, staple foods such as corn and rice are grown for self-consumption while fruits and vegetables are produced as cash crops using water from a river which crosses the two villages [18]. The dry season corresponds to the lean season, when the population depends mainly on stored grains and small livestock for their subsistence. During the rainy season, stagnant water promotes the breeding of mosquitos, the vector of malaria parasites. Malaria is endemic in the region; however, its incidence increases from June through October. In addition to malaria, diarrheal diseases and respiratory infections are the main reasons for medical consultation and hospitalization of children under 5 years of age in this area. Primary health care is provided at a primary health facility called “Centre de Santé et de Promotion Sociale (CSPS)”.

**Fig. 1:**
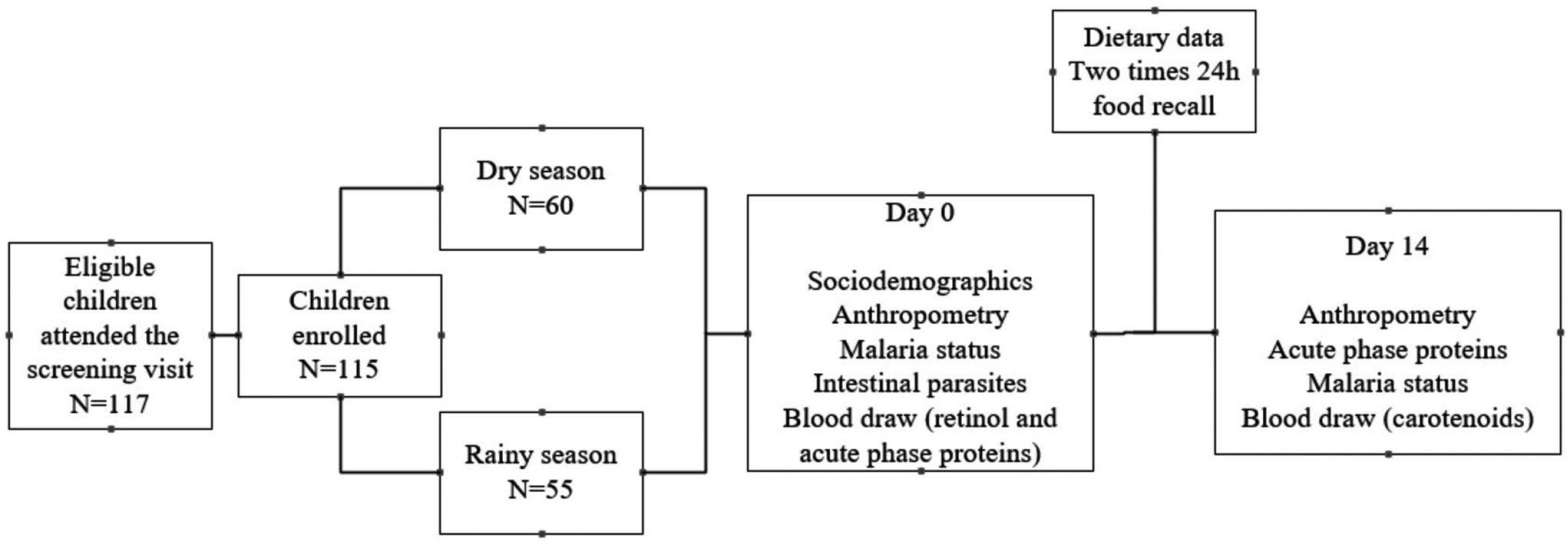
Schematic diagram of the study flow and data collection tools

The study included 115 apparently healthy children, from both sexes, 36–59 months of age who live in one of the two villages of Sourkoudougou and Banakeledaga. After a sensitization of the two communities, all participants and their caregivers were invited to come to the health center of Sourkoudougou for screening, enrollment and follow up activities. Children were enrolled in two separate surveys; the first one was conducted during the dry season from November 2016 to January 2017, and the second survey was carried out during the rainy season between August and September 2017 (Fig. 1). Children were included in the study after obtention of a written informed consent from one or both their parents. Participants whose birthdate could not be determined and those who: (1) were diagnosed with clinical signs of VAD including night blindness, conjunctival xerosis, Bitot’s spots, or corneal xerosis, ulceration or scars; (2) were severely acute malnourished (weight-for-height z-score [WHZ] < -3 standard deviations [SD], according to the 2006 WHO Child Growth Standards [19]); (3) had severe anemia with hemoglobin concentration < 70 g/L [20]; (4) had fever or reportedly had fever in the previous 24 h (fever was defined as an uncorrected axillary temperature above 37.5 °C); or (5) suffered from serious illness that necessitates hospitalization including coma, clinical severe dehydration, severe vomiting, or severe respiratory illness, were excluded.

### Baseline data collection

Data were collected for eligible children during the first day of enrollment. Data included the child’s sex and age, father’s occupation, maternal education, marital status, and household assets including building material, radio, television, telephone, refrigerator, bicycle, and motorcycle. Dietary intake data and information on breastfeeding practices were collected using adapted 24-hour dietary recall questionnaire [21].

On the same day, children’s height and weight were measured in duplicate. Height, in cm, was measured to the nearest 0.1 cm using a portable length board (Seca 213, Hamburg, Germany), and weight, in kg was assessed with 50 g precision using an electronic balance (Seca 899, Hamburg, Germany). A third measurement was completed in case the two measurements differed by > 0.5 cm and by > 0.1 kg for height and weight, respectively. The mean of the two closest values was used in the analysis.

### Blood sample collection and ^13^C_2_-retinyl acetate administration

We have followed the standard operating procedures in assessing VA status using stable isotope techniques [15]. All the procedures were carried out at the health center of Sourkoudougou. Briefly, during the first day (D0), 6 ml of venous blood were collected in plain tubes by a trained phlebotomist. Blood samples were protected from light by covering the tubes with aluminum paper. The protected tubes were immediately stored in an ice box and transported the same day to the laboratory of the “Institut de Recherche en Sciences de la Santé” (IRSS, Bobo Dioulasso). On the same day of their reception, blood samples were centrifuged at 3000 rpm for 10 min with a Universal 320R centrifuge (Hettich Zentrifugen, D-78,532 Tuttlingen). Serum was transferred into brown 2 mL cryotubes (Eppendorf, Hamburg, Germany) under yellow light and stored at -80 °C. Shortly after the D0 venous blood draw, 1.0 µmol ^13^C_2_-retinyl acetate [22], dissolved in 211.6 µL food grade soybean oil, was administered orally using a positive displacement pipette (Gilson Microman E M250 E, 50–250 µL). After making sure that the children swallowed the oil containing the stable isotope, they were given 1 ml of unfortified vegetable oil and a fat-containing snack to improve absorption and asked to eat it immediately. On day 14 after dose administration, another venous blood sample was drawn and processed in the same way as described above for the venous blood sample drawn at baseline.

All the pre-dose and post-dose samples were transported on dry ice to the University of Wisconsin-Madison for serum retinol and ^13^ C-retinol analysis. Serum extracts of 100 µL were transported on dry ice to the VitMin Lab, Willstaett, Germany for analysis of RBP and inflammatory biomarkers.

### Serum retinol analysis

This study reports retinol analysis on all the participants from D0 (Fig. 1). Serum samples were processed following a previously described procedure with minor modifications as described elsewhere [23]. A volume of 100 µL serum sample was denatured with 150 µL of ethanol and 75 µL of C23-β-apo-carotenol was added as an internal standard. Compounds of interest were extracted three times with 500 µL hexanes. The three supernatant fractions were pooled and dried under nitrogen gas, reconstituted in 75 µL methanol:dichloroethane (75:25 vol:vol), and 3 µL injected into the Waters Acquity H-Class ultra-pressure liquid chromatograph (UPLC) system, under chromatography conditions that were reported in details elsewhere [24].

### Total liver vitamin a reserves and total body stores

Method of extraction, purification, and analysis of ^13^ C using gas chromatography combustion isotope ratio mass spectrometry (GC/C/IRMS) was previously described [15, 25]. Briefly 0.3 to 1.0 mL serum sample was denatured with 2 ml of ethanol. C23 β-apo-carotenol was added as an internal standard. Retinol was extracted 3 times with 1–2 mL hexanes. The supernatant fractions were pooled, dried under nitrogen, resuspended in 100 µL methanol, frozen at -80˚C for 5 min, centrifuged at 1380 x g briefly, and injected into High-Performance Liquid Chromatography (HPLC) system 1 for quantification and purification. The retinol fraction, thus separated was collected, dried under nitrogen, and resuspended in 100 mL methanol for injection into HPLC system 2. The retinol fraction was dried in a Thermo Savant Speed-Vacuum centrifuge (Thermo Scientific, Waltham, MA), and reconstituted in 10 µl hexanes. Synthetic retinol, prepared by quick retinyl acetate saponification (Sigma-Aldrich, St. Louis, MO), was purified twice similarly to serum retinol and used as an external standard. Retinol extracts and the external standard (each 1.5 µl) were injected into the GC/C/IRMS with the programmed temperature vaporizing (PTV) injector. Atom percentage (At %) was directly calculated (Isodat version 2.0; Thermo Scientific) in reference to carbon dioxide, which was calibrated against a sucrose standard (National Institute of Standards and Technology, 8542).

Tracer-to-tracee ratio (TTR), which is an analogous to specific activity for isotopes was calculated using the following formula [26, 27]:

$${\text{TTR}}\,{\text{ = }}\frac{{{\text{Fc}} - {\text{Fb}}}}{{Fa - Fc}}$$

Where Fa is the ^13^C-abundance of the tracer dose [0.11 in ^13^C_2_- retinol (2 of 20 carbons labeled with ^13^C during synthesis [22] plus naturally abundant ^13^C at 1.1% in the remaining 18 carbons)]; Fb is the baseline abundance of ^13^C in retinol measured in the baseline serum sample D0; and Fc is the final ^13^C-abundance of serum retinol after the 14-d mixing period D14.

Total body VA stores (TBS, µmol) were calculated as previously described [25, 28] using the mass balance equation that includes the TTR:

$$TBS = a \times \frac{1}{{TTR}} \times (factors{\text{ }}for{\text{ }}absorption{\text{ }}and{\text{ }}storage)$$

Where *a* is the amount of ^13^C retinyl acetate in the dose (1.0 µmol) and the fraction of ^13^C to ^12^C in the dose is 0.11, which accounts for natural abundance. TLR (µmol/g) is calculated using the formula:

$$TLR = \left( {\frac{{TBS}}{{BW\left( {kg} \right)x\,liver\,fraction\,of\,BW}}} \right) \times 1000 \times fraction\,of\,TBS\,in\,liver$$

Where BW is body weight (kg), liver fraction of BW was estimated as 3% in preschool children, and 80% of TBS were assumed to be in the liver storage pool of children with adequate VA to hypervitaminosis [25, 28]. VAD and hypervitaminosis A were defined as TLR < 0.1 µmol/g of liver and TLR > 1.0 µmol/g of liver, respectively.

### Inflammation indicators and retinol binding proteins determinations

Acute phase proteins including CRP, AGP, and RBP were analyzed in serum samples by ELISA (DBS-Tech in Willstaett, Germany) [29]. The coefficients of variation (CVs) of the CRP, AGP and RBP for a pooled serum sample were respectively 5.84%, 8.09%, and 3.61%.

### Hemoglobin concentration, malaria and intestinal parasites

Using the remaining blood drops from the venous blood draw, hemoglobin concentration using point-of-care Hemocue (Hemocue HB 301, HemoCue® AB, Ängelholm, Sweden) was measured, and rapid diagnosis test (RDT; SD-Bioline Malaria-Ag-Pf/Pan™) for malaria [30] and malaria blood smear [31] were performed to assess malaria status. Thirty grams (30 g) of stool were collected on the spot from the children, and later a direct examination on a slide after preparation was performed by a qualified laboratory technician to search for the presence of intestinal parasites at the Centre Muraz (Bobo-Dioulasso). The test was considered negative for intestinal parasites if no parasite was identified.

### Data processing

Z-scores of height-for-age (HAZ), weight-for-age (WAZ) and weight-for-height (WHZ) were calculated using the WHO Child Growth Standards and the STATA macros [19]. Dietary diversity score ranging from 0 to 9, was constructed based on the reported child’s consumption of one of nine food groups during the 24-h dietary recall: starchy foods, dark green leafy vegetables, VA-rich fruits and vegetables, other fruits and vegetables, organ meat, meat and fish, eggs, legumes, nuts and seeds, and dairy products [32]. The household asset index was constructed based on the information on baseline ownership of a set of assets (radio, television, telephone, refrigerator, bicycle, or motorcycle) and building materials, using multiple correspondence analysis [33].

Vitamin A deficiency was defined by serum retinol or RBP concentrations < 0.7 µmol/L [6, 7]. Cutoffs of > 5.0 mg/L for CRP and > 1.0 g/L for AGP were used to define elevated acute phase proteins. Inflammation phases were defined as: (1) no inflammation if neither of acute phase proteins was elevated (CRP ≤ 5.0 mg/L and AGP ≤ 1.0 g/L), (2) incubation if CRP was elevated (CRP > 5.0 mg/L and AGP ≤ 1.0 g/L), (3) early convalescence if both CRP and AGP were elevated (CRP > 5.0 mg/L and AGP > 1.0 g/L), and (4) late convalescence when only AGP was elevated (CRP ≤ 5.0 mg/L and AGP > 1.0 g/L). Serum retinol and RBP concentrations were adjusted for the presence of inflammation based on elevation of one or both acute phase proteins (AGP and CRP) or no inflammation using the ratio between the arithmetic mean of the indicator in the respective inflammation category and its mean in the reference group, that is with no inflammation [13, 34]. Hemoglobin concentrations below 11.5 g/dL categorize children as anemic [20].

### Data analysis

All statistical analyses were carried out using Stata software, version 15.1 (Stata Corp, TX, USA). Descriptive statistics [geometric mean (95% confidence interval, 95% CI), means (± standard deviation, SD), and proportions] were performed to assess VA status, inflammation indicators and baseline sociodemographic and socio-economic characteristics and dietary intake.

Additionally, the accuracy of serum retinol and RBP were evaluated by calculating the sensitivity [True Positive / (True Positive + False Negative)], specificity [True Negative / (False Positive + True Negative)], positive predictive value [True Positive / (True Positive + False Positive)], and negative predictive value [True Negative / (False Negative + True Negative)] using TLR as the reference standard indicator. The diagnosis accuracy as a proportion of correctly classified subjects ([True Positive + True Negative] among all subjects) was also calculated [35].

With:

True positive: subjects with serum retinol or RBP concentrations < 0.7 µmol and TLR < 0.1 µmol /g of liver.

False positive: subjects with serum retinol or RBP concentrations < 0.7 µmol and TLR > 0.1 µmol /g of liver.

True negative: subjects with serum retinol or RBP concentrations > 0.7 µmol and TLR > 0.1 µmol /g of liver.

False negative: subjects with serum retinol or RBP concentrations > 0.7 µmol and TLR < 0.1 µmol /g of liver.

The effects of season, inflammation (CRP, AGP) and helminth infection on sensitivity and specificity were also assessed by estimating sensitivity and specificity in the presence versus absence of inflammation or helminth infection, and during dry season versus rainy season after participants classification in the above categories (true positives, false positives, true negatives, and false negatives).

## Results

### Baseline characteristics of participating children

This study reports on children who completed the protocol and have data on serum retinol concentrations, or RBP concentrations and TLR. Only 84 children had both serum retinol concentrations and TLRs, while 91 participants had both RBP and TLR. Slightly more than half of the children (53.9%) were male, and they were (mean ± SD) 48.9 ± 6.8 months of age. The most predominant activity of children’s fathers was farming (94.8%) and 79.1% of mothers did not have any formal education. Almost half (49.6%) of children were living in poor economic conditions. The prevalence of severe stunting (HAZ < -3 SD) was 4.4%. However, none was severely underweight (WAZ < -3 SD) nor severely wasted (WHZ < -3 SD), in this study sample. Intestinal parasites, malaria, high CRP, high AGP and anemia were diagnosed in 47.6%, 4.4%, 1.9%, 28.6%, and 14.9% of participants respectively. The mean dietary diversity score (± SD) was 4.1 ± 1.1 food groups. Table 1 summarizes the main sociodemographic and socioeconomic characteristics of the study participants.

**Table 1:** Baseline characteristics of participating children 36–59 months of age in rural Burkina Faso

Parameters^1^	Category	Study population *N* = 115
Child’s sex, male		62 (53.9)
Child’s age (month)		48.9 ± 6.8
Mother’s education	None	91 (79.1)
	Primary	17 (14.8)
	Secondary	5 (4.4)
	Other	2 (1.7)
Father’s occupation	None	2 (1.7)
	Farmer	109 (94.8)
	trader	1 (0.9)
	Other	3 (2.6)
Anthropometric indices	HAZ	-1.21 ± 1.08
	WHZ	-0.09 ± 0.92
	WAZ	-0.78 ± 0.80
Absence of morbidity^2^		113 (98.3)
Hemoglobin concentration (g/dL)		12.2 ± 1.4
Anemia	Non-anemic (Hb ≥ 11.5 g/dL)	97 (85.1)
	Mildly anemic (Hb < 11.5 g/dL)	17 (14.9)
Malaria	Positive malaria smear	5 (4.35)
	Positive RDT	6 (5.22)
Intestinal parasites	Positive slides	50 (47.6)
Acute phase proteins	CRP^3^	0.44 ± 5.71
	High CRP (> 5.0 mg/L)	2 (1. 9)
	AGP^3^	0.89 ± 0.52
	High AGP (> 1.0 g/L)	30 (28.6)
Dietary diversity score^4^		4.1 ± 1.1
Asset index		0.10 (-0.57; 0.58)

HAZ, height-for-age z-score; WHZ, weight-for-height z-score; WAZ, weight-for-age z-score; Hb, hemoglobin concentration

^1^ Values presented are means ± SD, *n* (%), and median (Q25- Q75)

^2^ Absence of clinical illness including cough, runny nose, and reported vomiting

^3^ Values were first log-transformed for analysis

^4^ Number of food groups consumed out of nine

### Vitamin A status biomarkers

**Fig. 2:**
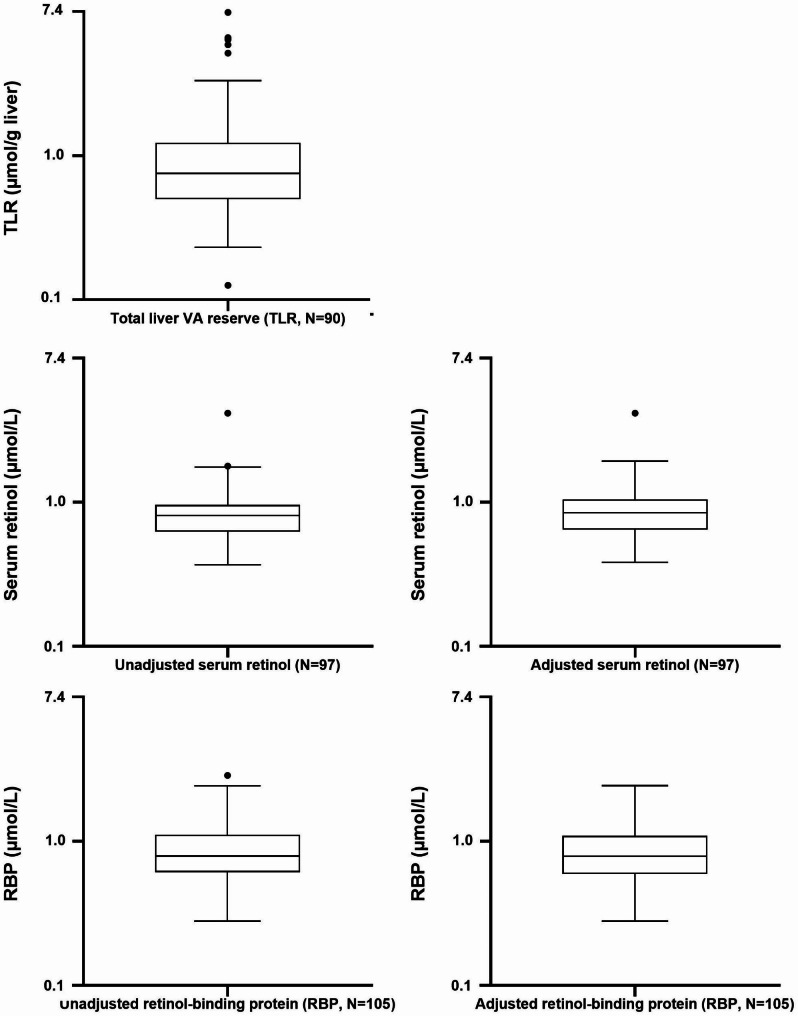
Total liver VA reserve (TLR), and unadjusted and adjusted serum concentrations in retinol, retinol binding proteins (RBP) of participating children 36–59 months of age

Vitamin A biomarkers included serum retinol, RBP, and TLR. Geometric means (95% CI) of TLR, and serum retinol and RBP concentrations were respectively 0.86 (0.75; 0.99) µmol/g liver, 0.82 (0.77; 0.88) µmol/L, and 0.83 (0.77; 0.89) µmol/L. Geometric means (95% CI) of serum retinol and RBP concentrations after adjustment for the presence of inflammation were 0.86 (0.81; 0.92) µmol/L and 0.81 (0.75; 0.87) µmol/L, respectively (Fig. 2).

Prevalence of VAD using unadjusted serum retinol and RBP was 30.9% (95%CI: 22.4; 40.9) and 33.3% (95%: 24.9; 43.0), respectively. After adjustment for inflammation indicators, the prevalence of VAD was 26.8% (95%: 18.8; 36.6) and 37.1% (95%: 28.4; 46.9), using serum retinol and RBP, respectively (Table 2). No VAD was detected by TLR estimations.

**Table 2:** Prevalence of vitamin A deficiency and accuracy of vitamin A biomarkers in children 36–59 of age in rural Burkina Faso

Parameters	Unadjusted serum retinol^1^	Adjusted serum retinol^1^	Unadjusted RBP^2^	Adjusted RBP^2^
Prevalence of VAD	30.9 (22.4; 40.9)	26.8 (18.8; 36.6)	33.3 (24.9; 43.0)	37.1 (28.4; 46.9)
Sensitivity^3^	-	-	-	-
Specificity^4^	71.1	75.9	68.9	67.8
Positive predictive value^5^	0	0	0	0
Negative predictive value^6^	100.0	100.0	100.0	100.0
True positive	0	0	0	0
False positive	28.9 (20.1; 39.7)	24.1 (16.0; 34.6)	31.1 (22.3; 41.5)	32.2 (23.3; 42.7)
False negative	0	0	0	0
True negative	71.1 (60.3; 79.9)	75.9 (65.4; 84.0)	68.9 (58.5; 77.7)	67.8 (57.3; 76.7)
Diagnosis accuracy^7^	71.1 (60.3; 79.9)	75.9 (65.4; 84.0)	68.9 (58.5; 77.7)	67.8 (57.3; 76.7)

VAD, Vitamin A deficiency; RBP, Retinol binding-protein; TLR, Total liver vitamin A reserves

Values are presented as % (95% Confidence Interval)

^1^*n*=97 for prevalence, and *n* = 83 for accuracy test

^2^*n*=105 for prevalence, and *n* = 90 for accuracy test

^3^Sensitivity [True Positive / (True Positive + False Negative)]

^4^Specificity [True Negative / (False Positive + True Negative)]

^5^Positive predictive value [True Positive / (True Positive + False Positive)]

^6^Negative predictive value [True Negative / (False Negative + True Negative)]

^7^Diagnosis accuracy [True Positive + True Negative]

### Accuracy of VA biomarkers in assessing VAD

Table 2 summarizes the test accuracy for detecting VAD per indicator used. Specificity and diagnosis accuracy of unadjusted serum retinol was 71.1%, suggesting that in this study, serum retinol identified correctly 71.1% of the population without VAD. However, 28.9% of participants were found to have VAD when they did not. Using the adjusted serum retinol concentration for inflammation status, false positives were reduced, increasing specificity to 75.9%. Adjusted RBP detected the highest prevalence of VAD. However, the highest specificity was found with adjusted serum retinol concentration. The adjusted RBP detected the highest number of false positives. The adjusted serum retinol diagnosed more accurately true negative.

Sensitivity was indeterminable (could not divide by zero) for serum retinol and RBP, because no case of VAD was detected using TLR estimations (i.e., true positive). Adjusted serum retinol for inflammation has the highest diagnosis accuracy (75.9%). On another hand, the lowest diagnosis accuracy (67.8%) was found with the RBP adjusted for inflammation.

### Factors influencing VA biomarkers accuracy in assessing VAD

The prevalence of VAD was higher using unadjusted vs. adjusted serum retinol, and during the dry vs. rainy season. The pattern could not be assessed in the presence of CRP because of the small number of participants with CRP ≥ 5.0 mg/L. While the prevalence of VAD was higher among children who did not have helminth infection (Table 3). On the contrary serum to retinol, the prevalence of VAD was higher using adjusted vs. unadjusted RBP. VAD prevalence was higher during the dry vs. rainy season, in the presence of CRP ≥ 5.0 mg/L, in AGP < 1.0 g/L, and in the absence of helminth infections (Table 4).

**Table 3:** Factors influencing serum retinol accuracy in the assessment of VAD in children 36–59 month of age in rural Burkina Faso

Parameters	Season		CRP		AGP		Helminth infections	
	Dry *n* = 40	Rainy *n* = 43	< 5.0 mg/L *n* = 79	≥ 5.0 mg/L *n* = 4	< 1.0 g/L *n* = 58	≥ 1.0 g/L *n* = 25	Negative *n* = 40	Positive *n* = 34
**Unadjusted serum retinol**								
Prevalence of VAD^1^	48.9 (34.7; 63.3)	14.0 (6.7; 27.0)	32.2 (23.5; 42.5)	-	29.4 (19.7; 41.5)	34.5 (19.1; 53.9)	39.1 (25.9; 54.2)	24.4 (13.4; 40.2)
Sensitivity^2^	-	-	-	-	-	-	-	-
Specificity^3^	52.5	88.4	69.6	-	74.1	64.0	60.0	79.4
Diagnosis accuracy^4^	52.5 (36.8; 67.7)	88.4 (74.4; 95.2)	69.6 (58.5; 78.9)	100	74.1 (61.1; 83.9)	64.0 (42.9; 80.8)	60.0 (43.8; 74.2)	79.4 (61.9; 90.1)
**Adjusted Serum retinol**								
Prevalence of VAD^1^	42.5 (29.0; 57.3)	12.0 (5.4; 24.6)	28.0 (19.7; 38.0)	-	29.4 (19.7; 41.5)	20.7 (9.3; 40.0)	32.6 (20.4; 47.7)	21.9 (11.6; 37.6)
Sensitivity^2^	-	-	-	-	-	-		
Specificity^3^	60.0	90.7	74.7	-	74.1	80.0	67.5	82.4
Diagnosis accuracy^4^	60.0 (43.4; 74.2)	90.7 (77.2; 96.6)	74.7 (63.8; 83.2)	100	74.1 (61.1; 83.9)	80.0 (58.8; 91.8)	67.5 (51.2; 80.4)	82.3 (65.1; 92.1)

CRP, C-reactive protein; AGP, alpha 1-acid glycoprotein; VAD, Vitamin A deficiency; RBP, Retinol binding-protein; TLR, Total liver vitamin A reserves

Values are presented as % (95% Confidence Interval)

^1^Dry season, *n* = 47; Rainy season, *n* = 50; CRP < 5 mg/L, *n* = 93; CRP ≥ 5.0 mg/L, *n* = 4; AGP < 1.0 g/L, *n* = 68; AGP ≥ 1.0 g/L, *n* = 29; No helminth, *n* = 46; Presence of helminths, *n* = 41

^2^Sensitivity [True Positive / (True Positive + False Negative)]

^3^Specificity [True Negative / (False Positive + True Negative)]

^4^Diagnosis accuracy [True Positive + True Negative]

**Table 4:** Factors influencing retinol binding proteins accuracy in the assessment of VAD in children 36–59 months of age in rural Burkina Faso

Parameters	Season		CRP		AGP		Helminth infections	
	Dry *n* = 47	Rainy *n* = 43	< 5.0 mg/L *n* = 86	≥ 5.0 mg/L *n* = 4	< 1.0 g/L *n* = 65	≥ 1.0 g/L *n* = 25	Negative *n* = 45	Positive *n* = 36
**Unadjusted retinol-binding protein**								
Prevalence of VAD^1^	42.6 (29.9; 56.3)	23.5 (13.7; 37.4)	32.7 (24.2; 42.5)	50.0 (4.0; 96.0)	37.3 (27.0; 48.9)	23.3 (11.2; 42.4)	33.3 (21.6; 47.6)	29.5 (17.7; 44.9)
Sensitivity^2^	-	-	-	-	-	-		
Specificity^3^	61.7	76.7	69.8	50.0	64.6	80.0	68.9	72.2
Diagnosis accuracy^6^	61.7 (46.8; 74.7)	76.7 (61.4; 87.2)	69.8 (59.1; 78.6)	50.0 (4.0; 96.0)	64.6 (52.1; 75.4)	80.0 (58.8; 91.8)	68.9 (53.6; 80.9)	72.2 (55.0; 84.7)
**Adjusted retinol-binding protein**								
Prevalence of VAD^1^	44.4 (31.6; 58.1)	29.4 (18.3; 43.6)	38.6 (29.5; 48.5)	0	38.7 (28.2; 50.3)	33.3 (18.5; 52.5)	37.2 (24.9; 51.5)	36.4 (23.3; 51.8)
Sensitivity^2^	-	-	-	-	-	-		
Specificity^3^	61.7	74.4	66.3	-	63.1	80.0	66.7	69.4
Diagnosis accuracy^4^	61.7 (46.8; 74.7)	74.4 (59.0; 85.5)	66.3 (55.5; 75.6)	100	63.1 (50.5; 74.1)	80.0 (58.8; 91.8)	66.7 (51.4; 79.1)	69.4 (52.2; 82.6)

CRP, C-reactive protein; AGP, alpha 1-acid glycoprotein; VAD, Vitamin A deficiency; RBP, Retinol binding-protein; TLR, Total liver vitamin A reserves

Values are presented as % (95% Confidence Interval)

^1^Dry season, *n* = 54; Rainy season, *n* = 51; CRP < 5.0 mg/L, *n* = 101; CRP ≥ 5.0 mg/L, *n* = 4; AGP < 1.0 g/L, *n* = 75; AGP ≥ 1.0 g/L, *n* = 30; No helminth, *n* = 51; Presence of helminths, *n* = 44

^2^Sensitivity [True Positive / (True Positive + False Negative)]

^3^Specificity [True Negative / (False Positive + True Negative)]

^4^Diagnosis accuracy [True Positive + True Negative]

The effects of season, inflammation and helminth infections on the specificity differed by the indicator used, and whether it was adjusted for inflammation or not (Tables 3 and 4). The specificity of unadjusted and adjusted serum retinol was lower during the dry than rainy one (52.5% vs. 88.4% and 60.0% vs. 90.7%, respectively), in CRP < 5.0 mg/L than CRP ≥ 5.0 mg/L (69.6% vs. 100% and 74.7% vs. 100%, respectively), and in the absence of helminth infections than in their presence (60.0 vs. 79.4% and 67.5% vs. 82.4%, respectively). The specificity of unadjusted-serum retinol was lower in AGP ≥ 1.0 g/L than in AGP < 1.0 g/L (64.0% vs. 74.1%), while specificity of adjusted-serum retinol has an opposite pattern (82.4% and 67.5%, respectively). Similar results were observed for specificity and diagnosis accuracy for unadjusted and adjusted RBP for season, AGP and helminth infections. The specificity of unadjusted and adjusted RBP was higher in AGP ≥ 1.0 g/L than in AGP < 1.0 g/L.

## Discussion

This study assessed the sensitivity, specificity, positive predictive value and negative predictive value of serum retinol and RBP concentrations, unadjusted or adjusted for inflammation status, to detect VAD among children using TLR determined by the RID test as the reference standard indicator. None of the children was VA deficient as indicated by their TLR values, while at least a third of the children were VA deficient using the adjusted or unadjusted serum retinol and RBP concentrations. The latter defines VAD in the study area as a serious public health concern based on WHO cutoff [10]. Similar findings were reported in preschool children exposed to inflammation in Zambia and Thailand, where respectively, 17% and 65% of children had VAD using serum retinol and 0% according to TLR [12]. It is noteworthy that TLR is considered a gold standard in assessing VA status. However, our analysis on the association between VA status, inflammation, and infections in children 36–59 months of age in rural Burkina Faso, found that TLR and TBS were associated with asymptomatic acute inflammation status and presence of intestinal parasites [36].

Only few studies have examined the sensitivity and specificity of serum retinol or RBP concentrations to determine VAD using as reference test, the MRDR [37, 38] and the RID [12]. The specificity of 71.1% for serum retinol and its improvement after adjusting for inflammation to 75.9% suggests the decrease in the number of false positives. This is consistent with the finding of previous studies in preschool children in Zambia, Thailand and Ghana, that reported an improvement in specificity after adjustment for inflammation indicators [12, 37]. Specificity improvement could be explained by the bidirectional relationship between infection and VA status, suggesting that infection negatively affects serum retinol [13, 39]. This pattern was less evident with RBP, whose specificity did not differ greatly after adjustment for inflammation. Our results differ from a similar study conducted in Ghana, that reported an improved specificity of RBP in detecting VAD among children exposed to inflammation after adjustment [37].

Specificity and diagnosis accuracy were low during the dry season compared to the rainy season, which is the period of high prevalence of infection in the study area such as malaria and diarrhea. Inflammation and infections decrease serum retinol and RBP concentrations, resulting in increased VAD prevalence or false positive. Adjustment for inflammation increases the specificity of both tests, as found in other studies using the MRDR [8, 11, 12]. However, it could be that adjusting for inflammation solely is not sufficient to capture the asymptomatic inflammation and infections, that children are exposed to, especially in malaria endemic areas. Previously, Wessels and co-authors reported the necessity to adjust for malaria in reporting VA status [17]. The low prevalence of children who had elevated CRP and AGP concentrations could have also affected the adjustment of VA markers in our study. Additionally, we have reported in another study that children who tested positive for the presence of intestinal parasites had higher TLR and TBS than children who tested negative [36], making also the reference test questionable.

RBP was considered as a good indicator of VA status in few studies using MRDR as reference test [40–42]. This is especially of importance considering that RBP is not light-sensitive like retinol and thus, easier to assess in large surveys in low- and middle-income countries. In the current study, specificity and diagnosis accuracy were similar to those obtained with serum retinol. This is in line with other studies in Zambia and Thailand [12].

The relative high prevalence of false positives in the estimation of VAD found with serum retinol and RBP, in addition to the high prevalence of children with hypervitaminosis A (> 1.0 µmol/g liver) calls for careful consideration and a proper evaluation of VA status at population level in Burkina Faso for designing national policy of VA.

This study has several strengths including the study design, the sample size and the variety of methods used to assess VA status. However, the study also is not without limitations which include the low number of children with asymptomatic malaria or with acute inflammation (CRP). Additionally, the subgroup analyses were performed by indicator (season, AGP, CRP and helminth infections). A complete model that can integrate all the indicators and define optimal conditions for an improved accuracy of determining VAD was not possible for the reference method did not detect any VAD (sensitivity could not be calculated).

## Conclusion

In children 36–59 months, no cases of VAD were identified by TLR in this study. However, serum retinol and RBP, unadjusted and adjusted for inflammation, had varying accuracy in the estimation of VAD prevalence. Specificity is high for all the VA markers. This specificity was variously affected depending on the participant’s inflammation status, the presence of intestinal parasites and the season of the year. The sensitivity was indeterminable. Prevalence of hypervitaminosis A is high, which calls for attention from programmers and policy makers regarding the various ongoing programs targeting VAD. Further work is needed to best understand the relationship between the VA biomarkers (serum retinol and RBP and their adjustment for inflammation) commonly used in large scale programs and on the potential health consequences of elevated TLR in population exposed to multiple VA interventions.

## Data Availability

Data used in this analysis are available at: https://osf.io/vrquy.

## List of abbreviations

AGP: Alpha 1-acid glycoprotein
At: Atom percentage
CRP: C-reactive protein
CSPS: Centre de Santé et de Promotion Sociale
GC/C/IRMS: Gas chromatography combustion isotope ratio mass spectrometry
HAZ: Height-for-age z-score
HPLC: High-Performance Liquid Chromatography
IRSS: Institut de Recherche en Sciences de la Santé
LMICs: Low- and middle-income countries
RBP: Retinol-binding protein
RDR: Relative dose response
RDT: Rapid diagnosis test
RID: Retinol isotope dilution technique
SR: Serum retinol
TBS: Total body vitamin A stores
TLR: Total liver vitamin A reserve
TTR: Tracer-to-tracee ratio
UPLC: Ultra-pressure liquid chromatograph
VA: Vitamin A
VAD: Vitamin A deficiency
WAZ: Weight-for-age z-score
WHO: World Health Organization
WHZ: Weight-for-height z-score
